# Exploring the relationship between effort perception and poststroke fatigue

**DOI:** 10.1212/WNL.0000000000010985

**Published:** 2020-12-15

**Authors:** William De Doncker, Lucie Charles, Sasha Ondobaka, Annapoorna Kuppuswamy

**Affiliations:** From the Department of Clinical and Movement Neuroscience, (W.D.D., S.O., A.K.) Institute of Neurology, and Institute of Cognitive Neuroscience (L.C., S.O.), UCL, London, UK.

## Abstract

**Objective:**

To test the hypothesis that poststroke fatigue, a chronic, pathologic fatigue condition, is driven by altered effort perception.

**Methods:**

Fifty-eight nondepressed, mildly impaired stroke survivors with varying severity of fatigue completed the study. Self-reported fatigue (trait and state), perceived effort (PE; explicit and implicit), and motor performance were measured in a handgrip task. Trait fatigue was measured with the Fatigue Severity Scale-7 and Neurologic Fatigue Index. State fatigue was measured with a visual analog scale (VAS). Length of hold at target force, overshoot above target force, and force variability in handgrip task were measures of motor performance. PE was measured with a VAS (explicit PE) and line length estimation, a novel implicit measure of PE.

**Results:**

Regression analysis showed that 11.6% of variance in trait fatigue was explained by implicit PE (*R* = 0.34; *p* = 0.012). Greater fatigue was related to longer length of hold at target force (*R* = 0.421, *p* < 0.001). A backward regression showed that length of hold explained explicit PE in the 20% force condition (*R* = 0.306, *p* = 0.021) and length of hold and overshoot above target force explained explicit PE in the 40% (*R* = 0.399, *p* = 0.014 and 0.004) force condition. In the 60% force condition, greater explicit PE was explained by higher force variability (*R* = 0.315, *p* = 0.017). None of the correlations were significant for state fatigue.

**Conclusion:**

Trait fatigue, but not state fatigue, correlating with measures of PE and motor performance, may suggest that altered perception may lead to high fatigue mediated by changes in motor performance. This finding furthers our mechanistic understanding of poststroke fatigue.

Fatigue after stroke, sometimes years after stroke, is prevalent, yet little is known about its underlying mechanisms.^1^ We proposed a sensory attenuation model of poststroke fatigue (PSF) in which poor sensory attenuation leads to heightened perception of effort, resulting in high fatigue.^2^

Perceived effort (PE) is believed to arise from a combination of top-down (expected) and bottom-up (actual) sensory inputs that encode muscle contraction. The jury is still out regarding the extent of relative top-down and bottom-up contributions to the experienced PE.^3–10^ The active inference framework of PE assumes that both top-down and bottom-up processes are gain modulated and that PE is a psychophysical output of the gain function.^11,12^ We proposed that in PSF heightened PE is driven by altered efferent (top-down) gain.^2^ There is no experimental evidence of altered PE in PSF. In this study, we provide evidence of heightened PE in PSF and put forward a rationale for how altered PE could result in PSF.

Reports of perception are subject to response bias arising from a number of sources.^13,14^ In a highly stigmatized and underrecognized^15,16^ condition such as PSF, response bias is tackled by introducing a novel implicit measure of PE. Visual perception is influenced by effort and line length perception^17,18^; a visual perceptual task is used as an implicit measure of PE.

Therefore, in this investigation we show that higher PE could result in high PSF, higher PE influences motor performance, and visual perceptual tasks can be used as a measure of PE.

## Methods

### Standard protocol approvals, registrations, and patient consents

The study was approved by London Bromley Research Ethics Committee (REC reference No. 16/LO/0714). Written informed consent was obtained from all patients in accordance with the Declaration of Helsinki.

### Participants

Patients with stroke were recruited via the Clinical Research Network from the University College NHS Trust Hospital, departmental Stroke Database, and the community. Four hundred thirty-six patients with stroke were contacted between January 2017 and June 2019. Patients were screened on the basis of the following criteria: (1) first-time ischemic or hemorrhagic stroke, (2) stroke at least 3 months before the study, (3) no other neurologic disorder, and (4) not taking antidepressants or other centrally acting medication. Patients who met the initial screening criteria were screened for a second time for the following: (5) depression scores ≤11 assessed with the Hospital Anxiety and Depression Scale, (6) no sensory impairment, and (7) grip strength and manual dexterity of the affected hand (at least 60% of unaffected hand) assessed with a handheld dynamometer and the Nine-Hole Peg Test (NHPT) respectively. One hundred thirty-two patients were interested in taking part in the study and met the initial screening criteria, of whom 113 met all eligibility criteria. To obtain a medium sized effect (*f*^2^ = 0.15) with statistical power of 0.8 at an α level of 0.05 using a single predictor, 54 datasets were deemed necessary. Fifty-eight patients with stroke took part in the experiment between January 2018 and June 2019. More patients were recruited than necessary to have at least 54 datasets that were eligible for final analysis. Self-reported fatigue was captured with the Neurologic Fatigue Index (NFI), a stroke-specific index,^19^ and the Fatigue Severity Scale 7 (FSS-7), which has been widely used and validated across different conditions.^20^

### Procedure

In this single-session cross-sectional study, patients performed a PE task on a desktop computer running Psychtoolbox (psychtoolbox.org) implemented within MATLAB (2016b, MathWorks, Natick, MA). Trait fatigue, a retrospective measure of fatigue based on recall, measuring the experience and effect of fatigue over 2 weeks leading up to the day of testing, was measured with 2 questionnaires (FSS-7 and NFI-Stroke). State fatigue, the momentary state of fatigue at the time and day of testing, was measured with the visual analog scale (VAS). PE was measured with a VAS and line length estimation, an explicit and implicit measure of PE, respectively. Written instructions were given to each patient before the start of the experiment.

### Line length familiarization

Patients were shown 6 lines: 3 belonged to the short category (1, 2, and 3 cm), and 3 belonged to the long category (10, 11, and 12 cm). After presentation of the 6 lines, patients were shown each of the learned lines without information about the category it belonged to, and were asked to judge the line length. Patients responded using the keyboard: left arrow key for short and right arrow key for long. They were then asked to rate their confidence in their response using a VAS. If patients' response was <100% correct, the procedure was repeated until they were able to distinguish between short and long lines.

### PE paradigm

**Figure 1:**
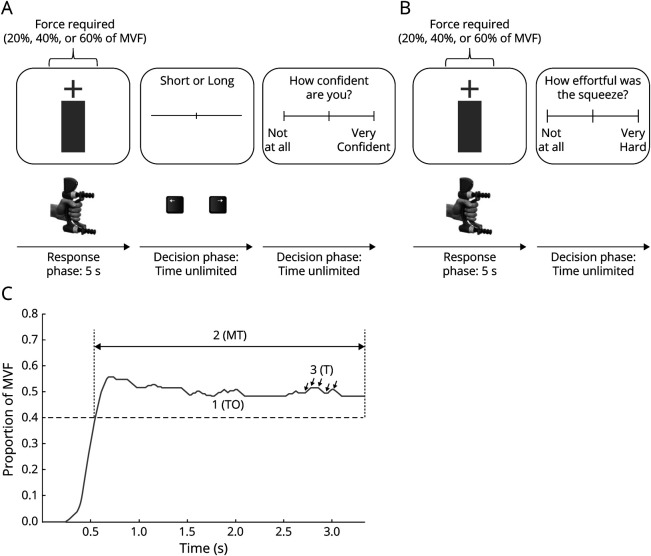
Task design Task design for (A) implicit perceived effort (PE) task and (B) explicit PE task (B). Each trial starts with a cross showing the target force level (20%, 40% or 60% maximum voluntary contraction [MVF]). Patients performed an isometric handgrip task using a handheld dynamometer and were instructed to get the bar to the target force level. (C) Example force trace with the 3 measures of motor performance (target overshoot [TO], length of hold [MT], force variability [T]). After 5 seconds, patients performed a line length estimation followed by confidence judgment in the implicit PE trials or explicitly reported how effortful the trial was in the explicit PE trials.

Patients sat facing a monitor (DELL 1909W, 19-in LCD display), held a handgrip dynamometer (Biometrics Ltd, Cwmfelinfach, Wales) with their dominant hand, and performed an isometric handgrip task. Force data from the dynamometer were acquired at 500 Hz via a data acquisition interface (Power1401, CED) and recorded in MATLAB (2016b, MathWorks). Each trial was 5 seconds long, in which patients were required to sustain a grip force for 3 seconds at 3 different levels: 20%, 40%, or 60% of their maximum voluntary force (MVF). Immediate force feedback was shown on the monitor as filling of a red bar, which turned green once the minimal required target force was reached. The minimal target force for each trial was indicated by a cross on the screen. The grip force–visual feedback relationship was individually adjusted for every patient to eliminate potential influence on PE. Before the experiment, patients practiced each force level with their dominant hand to familiarize themselves with the effort required. After each grip, participants performed a line length estimation. The line presented could have a length of 3.5 to 8.5 cm with a total of 24 different line lengths, 12 short and 12 long. Twenty-four lines presented under the 3 force conditions resulted in a total of 72 trials. The order of forces and line lengths was randomized with equal numbers of the 3 different force levels in each block. The experiment consisted of 3 blocks of 24 trials. Participants reported if the presented line was short or long by using the left and right arrow key of the keyboard, respectively. They were instructed to base their estimation on the length of lines presented during the familiarization phase. If they determined the presented line to be shorter than half the length of the longest line presented during the familiarization (12 cm), they reported short; otherwise, they reported long. There was no time limit on their response. They then rated their confidence using a VAS ranging from “not at all confident” to “very confident.” The intertrial interval was 1.5 seconds. The implicit PE task is shown in figure 1A.

After 3 blocks, participants performed a final block of 9 trials. This block was used as an explicit measure of PE (figure 1B). Each trial consisted of a 5-second grip with visual feedback at the 3 different force levels, 20%, 40%, or 60% of MVF, with 3 trials for each force level. This was followed by the question, “How effortful was the squeeze?” Patients had to respond using a VAS ranging from “not at all” to “very hard.”

### Analysis

Data were extracted from MATLAB into SigmaPlot (SigmaPlot Version 13.0) for statistical analysis.

### Fatigue questionnaires

Fatigue questionnaires were scored with the standard procedures in which the average of each of the 7 statement scores was considered the participant's overall fatigue score. FSS-7 is a scale of 1 to 7 with 7 being the highest fatigue and 1 being no fatigue. NFI is a scale of 0 to 3 with 3 being the highest fatigue and 0 being the lowest fatigue. FSS-7 was the primary fatigue scale; therefore, for all further analyses, fatigue scores refer to FSS-7 scores. The effect of FSS-7 on the patient demographics was examined with a Fisher exact test for categorical data and a Spearman correlation for continuous data.

### PE–explicit

VAS scores were averaged across the 3 trials in each force level (20%, 40%, and 60% MVF) for individual participants and were called VAS_20_, VAS_40_, and VAS_60_, respectively.

### PE–implicit

Two types of measures were extracted from the implicit PE trials. (1) A sum of the number of lines reported as long (SL) for each individual in each force level (SL_20_, SL_40_, and SL_60_ refer to number of lines in the 20%, 40%, and 60% MVF conditions). The total number of lines presented was 24 at each force level. Participants who reported all 72 lines to be long/short were excluded because this was taken as a failure to understand task instructions. Three participants were excluded on the basis of this criterion. (2) The proportion of long line responses categorized as long for each line length presented was calculated separately for each force condition in each participant. This measure allowed us to fit a psychometric curve for each participant in each force condition (the 20%, 40%, and 60% MVF), determining their sensitivity and bias in line length discrimination. From this fit, the sensory threshold (i.e., the point of equal proportion of response for each response option) and the sensory slope (defined as the inverse of the difference in line length observed between the point of 25% and 75% proportions of long line responses) were extracted. These measures were then compared across force conditions with paired *t* test (2 tailed) and correlated to fatigue score (FSS-7).

### Confidence

Confidence reports were made on a 0-to-10 VAS. To estimate the precision of confidence reports, a similar psychometric curve fitting approach to the proportion of response was used. The 0 to 10 confidence ratings were transformed according to the response so that a value of 0 would indicate maximal confidence in a short response and 1 would indicate a maximal confidence in a long response, 0.5 indicating the lowest confidence in both responses. The obtained confidence measure was averaged for each force condition, and a psychometric curve was fitted to the obtained average confidence. As for the previous analysis, the sensory threshold and sensory slopes of each participant in each force condition were extracted and correlated to fatigue scores.

### Motor performance and motor control

Three measures of motor performance were extracted from the isometric handgrip task for each MVF condition (20%, 40%, and 60%): (1) length of hold (MT), the maximal time in seconds spent above target in each trial, averaged across trials in each condition (MT_20_, MT_40_, MT_60_); (2) target overshoot (TO), the mean force exerted in each individual trial for the MT, expressed as a percentage of maximal force and averaged across trials in each condition (TO_20_, TO_40_, TO_60_); and (3) force variability (T), the number of transitions in the force trace during the time above target, counted and taken as the measure of T in each condition (T_20_, T_40_, T_60_). TO and MT were taken as motor performance parameters, and T was taken as a motor control parameter. A sample force trace of a single trial with the 3 measures of motor performance extracted is shown in figure 1C.

### Data availability

The data are available from the corresponding author (a.kuppuswamy@ucl.ac.uk) on reasonable request.

## Results

Fifty-eight patients with stroke (18 female, mean [SD] age 59.93 [12.44] years, mean [SD] time since stroke 4.23 [4.69] years) with mild physical impairment (mean [SD] grip score 91.03 [22.87], mean [SD] NHPT score 87.9 [23.21], mean [SD] Action Research Arm Test [ARAT] score 99.21 [3.42]) completed the study. FSS-7 and state fatigue scores ranged from 1 to 7 (total scale range 1–7) and 0 to 8 (total scale range 0–10) respectively. Patient demographics can be found in table 1. There was no significant difference in FSS-7 score on the basis of sex, hemisphere affected, or dominant hand and no significant correlation between FSS-7 score and age, time since stroke, grip, NHPT, ARAT, *Symbol Digit Modalities Test,* and anxiety score. There was a significant correlation between FSS-7 score and depression score, NFI score, and state fatigue (table 1).

**Table 1:**
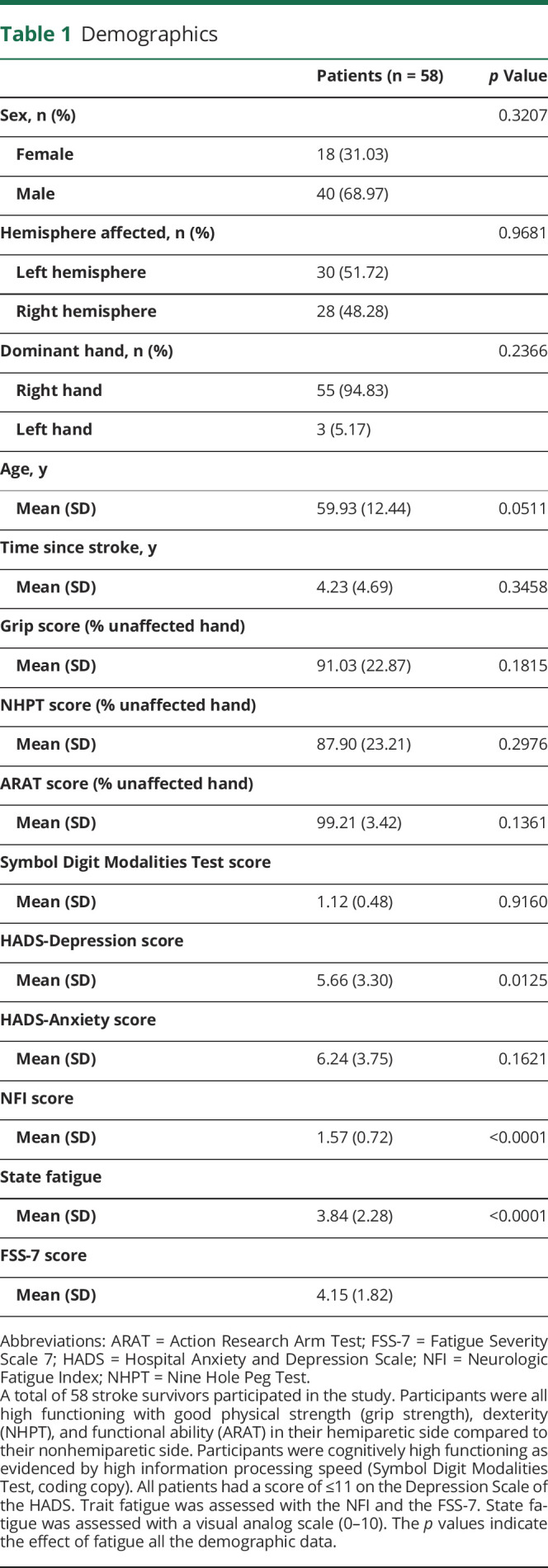
Demographics

### Collinearity analysis for PE and motor measures

A Pearson product-moment correlation analysis was performed between the explicit measures of PE (VAS_20,_ VAS_40,_ VAS_60_) and implicit measures of PE (SL_20,_ SL_40,_ SL_60_) in the 3 force conditions of 20%, 40%, and 60% maximal voluntary force. There were significant correlations between VAS_20_ and VAS_40_ (*r*[56] = 0.288, *p* = 0.03, confidence interval [CI] −0.27 to 3.44]) and between VAS_40_ and VAS_60_ (*r*[56] = 0.397, *p* = 0.002, CI 1.74–7.41) but not between VAS_20_ and VAS_60_. When considering the implicit measures of PE, we observed strong and significant correlations between SL_20_ and SL_40_ (*r*[53] = 0.869, *p* < 0.0001, CI 3.77–22.21), between SL_40_ and SL_60_ (*r*[53] = 0.868, *p* < 0.0001, CI 6.64–23.78), and between SL_20_ and SL_60_ (*r*[53] = 0.909, *p* < 0.0001, CI 6.46–24.03). Because the implicit measures of PE in the different force conditions (SL_20,_ SL_40,_ SL_60_) were strongly correlated, a combined measure of SL_sum_ was used in the final regression analysis whereby SL_sum_ was the sum of SL_20,_ SL_40_, and SL_60_. Despite significant correlations between some of the VAS scores, given the weak correlation coefficients, all 3 VAS measures were used as independent variables in the regression analyses. A Pearson product-moment correlation analysis was performed on the measures of TO (TO_20_, TO_40_, TO_60_), MT (MT_20_, MT_40_, MT_60_), and T (T_20_, T_40_, T_60_). There were strong and significant correlations between the 3 force levels in each of the measures TO, MT, and T. Table 2 shows the correlation coefficients and significance values of the pairs of conditions. Given the strong correlations, the 3 conditions in each motor parameter were averaged into TO_avg_, MT_avg_, and T_avg_ to be entered into the regression analysis for FSS-7 score.

**Table 2:**
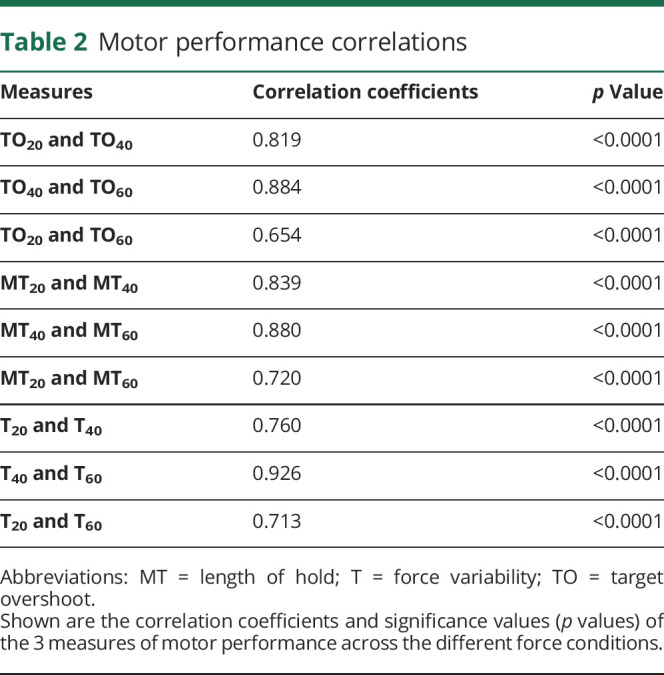
Motor performance correlations

### PE, confidence, and FSS-7 score

**Figure 2:**
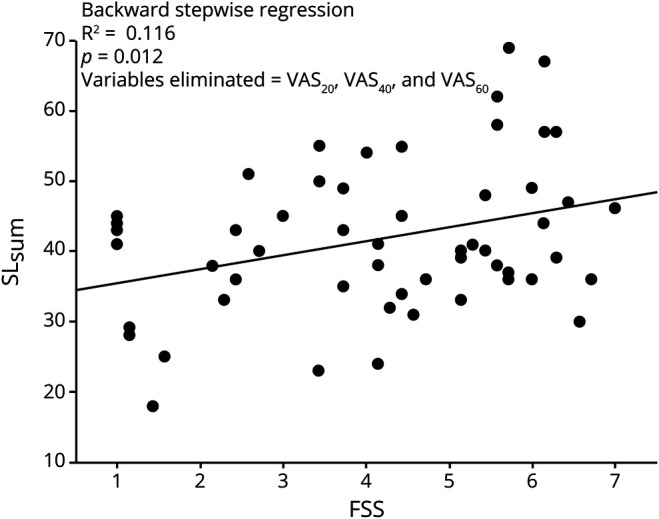
Effect of implicit PE on FSS-7 The combined measure of implicit perceived effort (PE) number of lines reported as long (SL_sum_; y-axis) is plotted against Fatigue Severity Scale 7 (FSS-7) score (x-axis). A significant correlation is seen between the 2 variables. VAS = visual analog scale.

**Figure 3:**
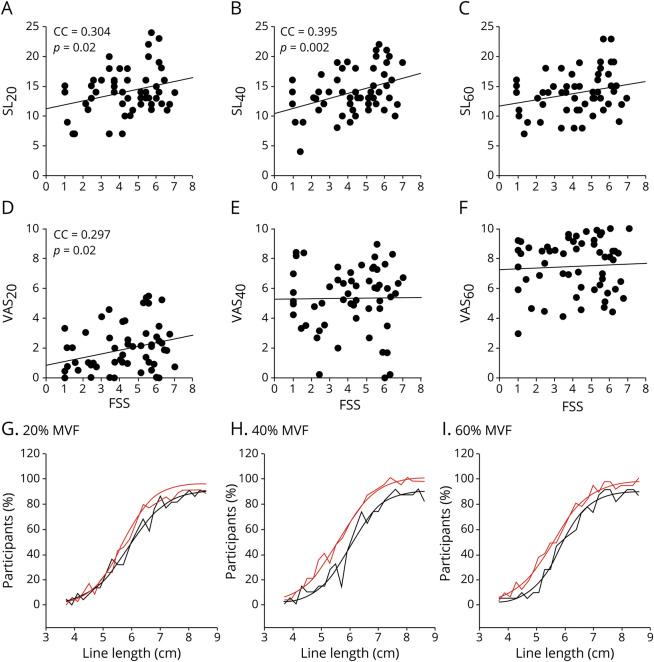
Implicit and explicit PE Measures of (A–C) implicit perceived effort (PE) and (D–F) explicit PE are plotted against Fatigue Severity Scale 7 scores (FSS-7; x-axis). There is a significant positive correlation between SL_20,_ SL_40_ and SL_60_ and FSS, with SL_20,_ and SL_40_ reaching statistical significance. There is also a statistically significant correlation between VAS_20_ and FSS-7 score but not in the higher force conditions. In this figure, the percent of participants (y-axis) who reported a given line length to be long is plotted against each line length (x-axis) presented during the implicit PE task (G–I) in the 3 different force conditions (20%, 40%, and 60% maximum voluntary force [MVF]). Red line represents the fatigue group with score >4 (n = 32); black line represents the fatigue group with score <4 (n = 22). Estimated midpoint of a 12-cm line is shifted to the left by 0.5 cm in all 3 force levels in the high fatigue group (red line) compared to low fatigue group (black line). CC = correlation coefficient; SL = number of lines reported as long; VAS = visual analog scale.

To test the effect of fatigue on PE, a stepwise backward linear regression analysis was performed with FSS-7 as the dependent variable and SL_sum_, VAS_20,_ VAS_40_, and VAS_60_ entered into the model as independent variables. Of the 4 measures of PE, SL_sum_ explained 11.6% of the variance in FSS-7 score (*R* = 0.34, *p* = 0.012, CI 1.69–7.02, figure 2), with the explicit VAS measures not contributing significantly to FSS-7 score. Figure 3, A–F plots all 6 measures of PE (VAS_20,_ VAS_40,_ VAS_60_, SL_20_, SL_40_, SL_60_) against FSS-7 scores. Figure 3, G–I shows the implicit PE measure when the cohort of 58 stroke survivors were divided into high (FSS-7 score >4) and low (FSS-7 score <4) fatigue. Although there was no significant difference in the estimated midpoint of the line length between the high and low fatigue groups, there was a consistent 0.5-cm leftward shift in all 3 force levels in the high fatigue group compared to the low fatigue group.

**Figure 4:**
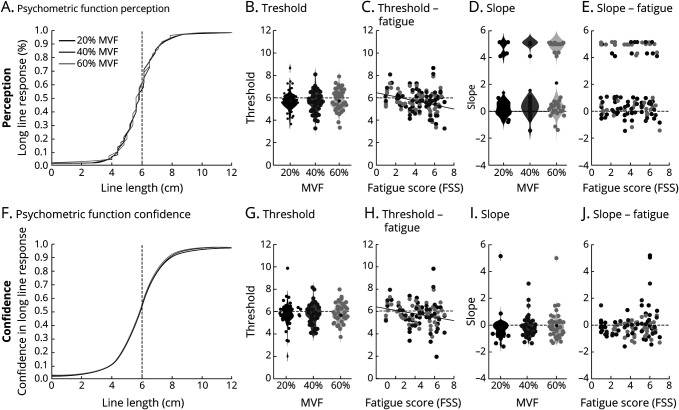
Effort perception and confidence Fitting of the psychometric curve for the line length discrimination task based on (A–E) response choice and (F–J) confidence ratings in each effort condition (dark gray = 20% maximum voluntary force [MVF]; medium gray = 40% MVF; light gray = 60% MVF). (A and F) Average fitted psychometric curve between presented line length and proportion of long responses or rescaled confidence ratings across participants. Each fit was performed separately for each force condition and each participant. (B, D, G, and I) Violin plots of the obtained fit parameters corresponding to the (B and G) sensory threshold and (D and I) sensory slope on the basis of (B–D) proportion of response or (G–I) confidence judgments for each condition. Black circle represents the population mean. (C, E, H, and J) Correlation results between Fatigue Severity Scale (FSS) scores and psychometric curve parameters according to (C and E) response choice or (H and J) confidence ratings for each effort condition. Significant correlations are indicated by a plain line.

**Figure 5:**
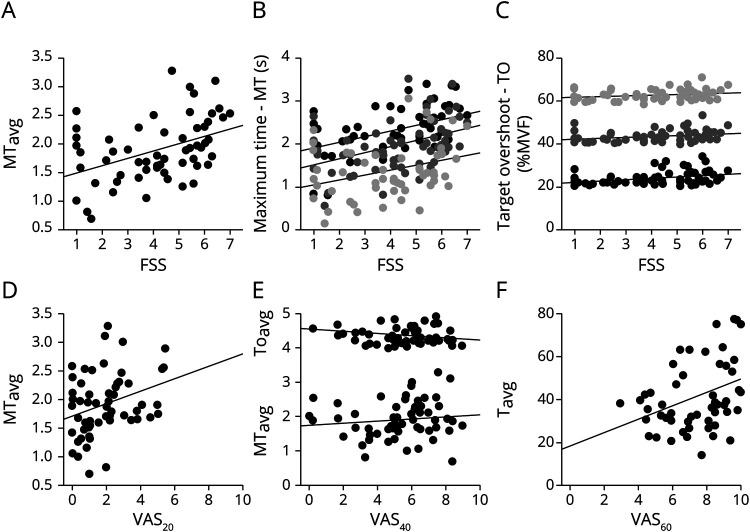
Motor performance and motor control Combined measure of time (seconds) above target (MT_avg_; y-axis) is plotted against Fatigue Severity Scale (FSS; x-axis). A small but significant correlation is seen between the 2 variables. In this figure, we plot TO_20_, TO_40_, TO_60_, MT_20_, MT_40_, and MT_60_ in the isometric hold task. Maximum time in seconds (MT; left) and target overshoot (TO) as percent maximum voluntary force (MVF; right) are plotted against FSS scores (x-axis). There is a statistically significant positive correlation between fatigue and both parameters of motor performance in all 3 force conditions. The relationship between explicit perceived effort (VAS_20_, VAS_40_ and VAS_60_) is plotted against parameters of motor performance (MT_avg_ and TO_avg_) and motor control (T_avg_). VAS_20_ and VAS_40_ are partially explained by regressors of motor performance, time above target (MT_avg_) and target overshoot (TO_avg_), while VAS_60_ is partially explained by measure of motor control–force variability (T_avg_). VAS = visual analog scale.

Figure 4, A and F shows the average curve fits for each line length presented and each force condition for the proportion of long responses (figure 4A) and the associated confidence in a long line response (figure 5F). For each participant, the fitted slope and sensory threshold were extracted and compared across conditions. Sensory threshold was significantly lower than 6 (midpoint) for force conditions above 40% MVF, when considering proportion of response (40% MVF: *t*[54] = −2.2, *p* = 0.032, CI 5.45–5.97]; 60% MVF: *t*[52] = −2.34, *p* = 0.023, CI 5.47–5.95) or confidence (40% MVF: *t*[54] = −2.15, *p* = 0.036, CI 5.48–5.98; 60% MVF: *t*[52] = −2.18, *p* = 0.034, CI 5.34–5.97), suggesting that participants were biased toward perceiving the line as longer than its actual length. However, sensory thresholds were not significantly different between force conditions when estimating it on the basis of the proportion of response (all *p* > 0.92) and confidence judgments (all *p* > 0.48). Similarly, no significant difference was found between force conditions when estimating the sensory slope on the basis of proportion of response (all *p* > 0.15) or on confidence report only (all *p* > 0.33). We then tested whether the psychometric curve parameters correlated with the individual fatigue scores, separately for each force condition and when all conditions were pooled together. No significant correlation was found between the sensory slope and fatigue scores for any of the conditions or when all conditions were pooled together, when considering the proportion of response (all *p* > 0.14) or confidence reports (all *p* > 0.5367), suggesting that perceptual sensibility was not affected by fatigue. A significant negative correlation was found, however, between fatigue and sensory threshold for the intermediate force condition 40% MVF, when considering both response proportion (*r*^2^ = 0.11, *F* = 6.61, *p* = 0.01, CI −0.32 to −0.03) and confidence reports (*r*^2^ = 0.09, *F* = 5.51, *p* = 0.02, CI −0.29 to −0.02). A similar trend was observed when considering the proportion of response and pooling all conditions together (*r*^2^ = 0.05, *F* = 3.17, *p* = 0.08, CI −0.25 to 0.01). This suggests that higher fatigue scores were associated with a stronger bias toward perceiving the lines as longer than they are.

### Motor performance, control, and FSS-7 score

To test how fatigue affected motor performance and motor control, a stepwise backward linear regression analysis was performed with FSS-7 score as the dependent variable and TO_avg_, MT_avg_, and T_avg_ entered into the model as independent variables. Of the 3 measures, MT_avg_ explained 17.8% of the variance in FSS-7 score (*R* = 0.421, *p* < 0.001, CI 1.4–7.25, figure 5A), with measures of TO and T not significantly adding to the explanatory power of the variables. Figure 5, B and C plots TO_20_, TO_40_, TO_60_, MT_20_, MT_40_, and MT_60_ against FSS-7 score, all of which significantly correlated with FSS-7 individually. The measure of T_20_, but not T_40_ and T_60_, correlated with FSS-7 score.

### Motor performance, control, and PE

Implicit PE (SL_sum_) was not explained by any of the motor performance and motor control measures. A stepwise backward linear regression analysis with explicit PE in the 20% force condition (VAS_20_) as the dependent variable and TO_avg_, MT_avg_, and T_avg_ as independent variables showed a small but significant contribution of MT_avg_ to VAS_20_ (*R* = 0.306, *p* = 0.021, CI −0.11 to 4.15) explaining 9.4% of the variance (figure 5D). Similarly, 15.9% of the variance in VAS_40_ was explained by a combined measure of MT_avg_ and TO_avg_ (*R* = 0.399, *p* = 0.014 and 0.004, CI −0.34 to 8.24, figure 5E), and T_avg_ explained 9.9% of the variance in VAS_60_ (*R* = 0.315, *p* = 0.017, CI 5.77–11.61, figure 5F).

### State fatigue, motor performance, motor control, and PE

All the above tests were performed with state fatigue as the dependent variable, and none of the measures of PE and motor measures explained the variance in state fatigue.

## Discussion

In this cross-sectional, observational study of 58 chronic, first time, nondepressed, mildly impaired stroke survivors, we show a significant relationship between fatigue and PE. In the absence of prior exertion, higher self-reported trait fatigue can be explained by a higher level of implicit PE observed during a physical task, while self-efficacy measures accurately reflected perceptual performance regardless of fatigue levels. We also show that explicit measure of PE fails to explain trait fatigue. Behaviorally, prolonged motor output indexed by longer sustained target forces was associated with high fatigue levels. Prolonged time above target and greater force than required were related to higher explicit PE in the low and medium force conditions. In the high force condition, greater PE was associated with higher T during the task. The measure of state fatigue was not explained by either altered PE or altered motor control and performance.

Fatigue in the chronic phase after stroke is related to lower motor cortex excitability and poor attention and is independent of motor weakness, lesion location, or biological markers of fatigue such as inflammation.^1,21,22^ On the basis of this wide range of findings in PSF, we proposed a framework wherein sensory information is incorrectly gated, possibly due to poor sensory predictions, leading to altered perception. Altered perception, specifically altered perception of effort in the context of motor actions, underpins PSF.^2^ Here, we show that the greater the fatigue in stroke survivors is, the higher the effort in a motor task is, despite individual calibration of task related force. This suggests, for the same proportional afferent input from the contracting muscle, that there is a greater sensory prediction error, giving rise to higher sense of effort in the individuals with high fatigue. An explanation of how PE is the psychophysical output of sensory prediction error is given elsewhere.^2^ Inaccurate sensory predictions and PE have been extensively studied in schizophrenia, in which a lack of effort in relation to movement leads patients to attribute movement to external control.^23^ The almost complete lack of predictions in schizophrenia has been shown to drive this sense of external control, by near abolition of PE.^24,25^ A similar fundamental framework of PE and sensory predictions can explain the result of higher PE. We propose that higher PE is driven by decreased gain on (less precise) sensory predictions as opposed to a lack of predictions seen in schizophrenia. Both explicit and implicit measures of PE correlated with trait fatigue in the lowest force level. However, in higher force levels, only implicit PE correlated with trait fatigue. A possible explanation is that explicit PE is not subject to response bias when the task is relatively easy, but with greater difficulty, response bias invalidates the measure of explicit PE. A second possible explanation is that the inability to consciously access accurate interoceptive information during high-effort activity in itself may drive the feeling of fatigue.

An interesting yet counterintuitive finding in this study is the significantly higher force levels and hold times exerted by those with high fatigue during the task. It is unclear why one must exert higher force when reporting high levels of fatigue. A possible explanation could be that those with higher fatigue have less precise sensory predictions, and hence, to ensure successful task completion, they exert greater force than required. Exerting greater force, and for longer, results in higher PE, as shown here in the low and medium force conditions. A recent study investigating the relationship between force and PE demonstrates that PE is not simply a function of metabolic cost. PE is a reflection of several movement-related cost parameters, of which time is a major driver i.e., the longer the motor performance, the higher the PE.^26,27^ Therefore, greater PE seen in this study could result from prolonged grip that is driven by poor sensory predictions. Such altered motor performance in fatigue has previously been reported in patients with cancer fatigue.^28^ Cancer survivors with high fatigue, not under medication, tended to opt for more high effort trials in an effort-based choice task. These results, taken together with the current study, point to a disease-independent motor behavior trait in high fatigue.

The corticospinal tract (CST) can be directly affected by stroke, and a question that arises is whether changes in output pathways may directly affect PE. First, in this study, we excluded those with moderate to severe muscle weakness, thereby ensuring minimal changes in CST and that any differences were similar across high and low fatigue. Despite this exclusion criterion, there is still a possibility of alterations in CST playing a role in driving greater force and longer holds in the grip task. Force variability is a measure of motor control and is consistently altered in those with stroke.^29^ In the current study, T does not explain the difference in PE in both the low and middle force conditions, suggesting that PE cannot be directly attributed to changes in efferent output pathways. However, in the high force condition, we see that greater T explains higher fatigue. This suggests that perception of effort is possibly informed by different movement parameters in the low force conditions compared to the high force conditions.

The results of the current study do not allow further elaboration. However, it would suffice to say that future studies into perception and PSF must carefully differentiate between high- and low-effort tasks. Establishing direction of causality between PE and fatigue is paramount if meaningful treatments are to be developed. As with many chronic symptoms, it is very difficult to establish the order of appearance of the various changes seen in performance and perception related to fatigue. A pertinent question that arises is whether fatigue can result in higher PE. The answer is invariably yes. However, the methodology used to capture fatigue may give us some room for nuanced interpretation of the current results. Trait and state measures of fatigue capture 2 very different phenomena. As with all affective measures, trait measures capture a more stable state of being, while state measures capture the momentary state of being. Trait measures may be influenced by state measures and vice versa. However, in this study, all performance and perceptual measures were performed at a single point in time, leading to state fatigue not always reflecting the trait measure. What this means is, in some patients, despite experiencing high levels of fatigue over a certain period of time past, the momentary state during performance of the laboratory tests was different. Here, it is a reasonable assumption that immediate perception and performance are likely to be influenced by the state of the being at that moment, i.e., by the state measure of fatigue, not the trait measure. The lack of a significant relationship between state fatigue and measures of perception and performance suggests that fatigue may not be driving perception and performance. However, to definitively establish that altered perception may drive fatigue, one must alter perception to see if a change in fatigue is observed. Future intervention paradigms must track perception to identify whether any change seen in fatigue is driven by altered perception.

The relationship between PSF and PE has been studied in a well-defined group, which limits its generalizability. However, we would consider this a strength of the study also in that it gives us a clearer picture of primary fatigue and its mechanisms. Future studies must include both depressed and nondepressed patients. The number of trials in each force–line length condition could be viewed as a limitation. Despite the 24 different line lengths, the estimated line length measure required participants to divide them into 2 categories: short or long. Therefore, there were 12 trials in the short category and 12 in the long category. Because the response required was 1 of 2 and not 12, for the estimated line length measure, effectively there were 12 repetitions in each force–line length combination. We chose not to have the exact line length for 12 repetitions because this could have resulted in a learning effect. Having any more than 12 in each condition would have considerably lengthened the protocol, resulting in fatigability playing a role in the results. The handgrip task was relatively simple with very little performance variability; hence, 12 trials were deemed sufficient for a representative average for each condition.

For the metacognitive measure of confidence, however, we used a continuous VAS scale of 0 to 10, and there could, in theory, be 24 different confidence levels for the 24 different line lengths. Hence, we must consider the experimental study design to have only 1 repetition of each force–line length combination for the confidence measure. The confidence data should therefore be interpreted cautiously.

We show that high implicit PE explains high PSF, possibly mediated by changes in motor performance. We also show that altered perception is not accompanied by reduction in self-efficacy. These results add strength to the idea that fatigue is driven predominantly by perceptual changes underpinned by sensory disturbances and that treatments must focus on modifying sensory processing and perception.

## Glossary

ARAT: Action Research Arm Test
CI: confidence interval
CST: corticospinal tract
FSS-7: Fatigue Severity Scale 7
MT: length of hold
MVF: maximum voluntary force
NFI: Neurological Fatigue Index
NHPT: Nine-Hole Peg Test
PE: perceived effort
PSF: poststroke fatigue
SL: number of lines reported as long
T: force variability
TO: target overshoot
VAS: visual analog scale
VAS_20_: explicit PE in 20% MVF condition
VAS_40_: explicit PE in 40% MVF condition
VAS_60_: explicit PE in 60% MVF condition
